# A Practical Treatise on the Diseases of Children

**Published:** 1853-07

**Authors:** 


*A Practical Treatise on the Diseases of Children.* By J. Forsyth Meigs, M. D., Second Edition, Revised and Enlarged. Philadelphia. Lindsay & Blakiston, 1853.

This work, which is well named a *Practical Treatise*, was originally published as one of the popular series of works, known as the Medical Practitioner's and Student's Library. The present edition is very considerably enlarged and greatly improved.

An introductory essay has been added on the clinical examination of children, a vitally important and very difficult piece of practice, as every medical man of any experience in the treatment of infantile diseases can testify. We cannot pass over this chapter without the remark that it is one of the very best accounts of the matter we have ever read. It is eminently practical, and contains hardly a sentence the teachings of which cannot be most advantageously used at the bedside of a sick child. It is just what the practitioner needs in his daily ministrations; it is exactly what the student wants to fit him for the duties which are soon to devolve upon him.

The introduction is a true type of the rest of the work. It is full, minute, and yet eminently practical. Where the entire task is so well done, it is difficult to particularize an individual merit. We were, however, much struck with the chapter on indigestion, a disease of children too little understood, and too apt to be confounded with others, and consequestly to be mismanaged in the treatment. The chapters on the exanthemata, and on that scourge of the poor little creatures, cholera infantum, are, as they should be, very full and satisfactory.

It is hardly necessary, after what we have already said, to make use of the stereotyped expression that we heartily commend the book to the notice of the profession, and yet we know of no work to which this phrase can be more truly applied. It is no mere abstract or compilation of the views of other men. While Dr. Meigs has availed himself, with commendable industry, of the labors of his predecessors, he embodies in this book the result of his own observations which have been numerous and very carefully made.

